# Characterization and Performance of Non-Activated Apricot Stone Powder for the Remediation of Zn^2+^-Rich Galvanizing Effluents

**DOI:** 10.3390/molecules31071143

**Published:** 2026-03-30

**Authors:** Aleksandra Nesic, Antonije Onjia, Milan Momcilovic, Jelena Maletaskic, Hao Dong, Shuai Chen

**Affiliations:** 1 Vinca Institute of Nuclear Sciences–National Institute of the Republic of Serbia, University of Belgrade, Mike Petrovica Alasa 12-14, 11001 Belgrade, Serbia; jelena.pantic@vin.bg.ac.rs; 2 Faculty of Technology and Metallurgy, University of Belgrade, Karnegijeva 4, 11000 Belgrade, Serbia; 3 Department of Chemistry, Faculty of Sciences and Mathematics, University of Niš, Višegradska 33, 18000 Niš, Serbia; milan.momcilovic@pmf.edu.rs; 4 School of Resources and Environmental Engineering, Shanghai Polytechnic University, Jinhai Road 2360, Pudong, Shanghai 201209, China; hao.dong@inv.uam.es (H.D.); chenshuai@sspu.edu.cn (S.C.)

**Keywords:** industrial effluents, food waste, heavy metal adsorption, wastewater purification, batch sorption kinetics, germination index

## Abstract

This study investigated the application of apricot stone, an agro-industrial by-product, as a sustainable biosorbent for the removal of Zn ions from aqueous solutions and industrial galvanic wastewater. The equilibrium data conformed well to the Sips isotherm model, indicating heterogeneous sorption behavior, and revealed a maximum sorption capacity of 58.2 mg/g. The biosorbent exhibited a high initial removal efficiency of 95% in aqueous Zn solutions, while its performance in real industrial wastewater was reduced to 55%, due to matrix interference. Ecotoxicological test using seed germination assays revealed no phytotoxic effects from the Zn-loaded sorbent. These findings demonstrate that apricot stone is an effective, low-cost, and environmentally friendly sorbent with significant potential for application in Zn-contaminated water treatment systems, contributing to circular economy and waste valorization initiatives.

## 1. Introduction

Zinc (Zn) is an essential micronutrient; however, its discharge into aquatic ecosystems at concentrations exceeding regulatory limits poses a significant environmental threat. Due to its non-biodegradability, Zn is subject to bioaccumulation and biomagnification, which can lead to systemic ecological toxicity [1]. Industrial effluents, particularly from the galvanizing and mining sectors, are a primary source of Zn contamination. These wastewaters are characterized by extreme pH levels and complex chemical matrices, making efficient treatment a technical challenge [2,3]. While conventional methods such as chemical precipitation and membrane technologies are widely used, they often incur high operational costs and generate secondary toxic sludge [4,5]. Consequently, adsorption has emerged as a superior alternative for the selective removal of Zn ions, offering high efficiency, cost-effectiveness, and the potential for utilizing sustainable biobased materials [6,7,8,9].

Over the past decade, biosorption using agricultural and food waste has emerged as a prominent strategy for heavy metal remediation [10,11,12,13]. This approach aligns with circular economy principles by transforming low-value fruit processing byproducts, such as peels and pulp, into high-efficiency sorbents, thereby mitigating the environmental burden of landfill disposal and greenhouse gas emissions. Among these waste streams, apricot stones (AS) are particularly noteworthy due to their lignocellulosic composition. Serbia is a leading producer in Southeast Europe, generating approximately 40,000 tons of apricots annually [14], which results in a significant volume of stones as a byproduct. While these stones are currently utilized in the cosmetic industry [15,16] or as precursors for activated carbon [17,18,19,20,21], research focusing on the direct application of raw apricot waste for heavy metal sequestration remains limited.

Despite the established use of AS for activated carbon production, the energy-intensive thermal activation processes required for such adsorbents often offset the economic and environmental benefits of using waste materials. This study addresses this gap by evaluating the raw lignocellulosic matrix of Serbian apricot stones as a sustainable, low-cost biosorbent for Zn(II) removal. By bypassing high-temperature carbonization, this approach maximizes the process’s ‘green’ credentials and economic efficiency, providing a practical solution for the final polishing of industrial wastewater. A thorough physical characterization of a raw apricot stone powder, evaluating its structural, textural, morphological, and crystallographic properties, has been conducted. Following this, the efficacy of raw, unmodified, and inactivated apricot stones as sorbents for Zn ions was assessed through batch sorption experiments in aqueous solution, systematically varying operational parameters. To complement this, the sorbent’s performance was rigorously tested using industrial wastewater from a galvanization plant. While prior research on apricot stone focused on Cu(II) and Cr(VI) removal, this study targets Zn(II) because its higher hydration energy and lower affinity for lignocellulosic ligands suggest a more challenging uptake mechanism [22,23]. Therefore, this work aims to investigate the specific efficiency and competitive behavior of Zn(II) in real wastewater systems to evaluate the material’s practical applicability.

Additionally, an ecotoxicological assessment of the Zn-loaded sorbent was conducted to determine its environmental impact. In general, raw agricultural-industrial waste, including apricot seeds and shells, used as sorbents often presents limitations such as low adsorption capacity and leaching of soluble organic compounds, prompting the investigation of various enhancement methods, including activation processes and pyrolysis [6,22]. The novelty of this study lies in the application of non-modified apricot stones to real industrial wastewater, integrated with an ecological assessment of the material after the sorption process.

## 2. Materials and Methods

Apricot stones (*Prunus armeniaca*) were obtained from fruits acquired from a local market in Serbia. The kernels were carefully separated and discarded, and the remaining shells were air-dried for two weeks. The dried shells were then milled, finely ground, and sieved through a 50 μm mesh. To facilitate the removal of impurities, organic-soluble matter, and polyphenols, the ground material underwent a 24 h water wash, followed by an ethanol wash. Zinc chloride salt (Sigma-Aldrich, St. Louis, MO, USA) was used for batch biosorption experiments. Wastewater containing Zn ions was obtained from the galvanization industry in Serbia, and its chemical composition is presented in Table 1.

### 2.1. Characterization of Apricot Stone Powder

The chemical structure of the apricot stone powder was investigated using a Thermo Nicolet 6700 Fourier Transform Infrared (FTIR) spectrometer (Thermo Fisher Scientific, Waltham, MA, USA), which acquired spectra over a frequency range of 400–4000 cm^−1^. The crystalline structure was analyzed using a Bruker D8 ADVANCE X-ray diffractometer (Bruker, Billerica, MA, USA). The instrument was operated with Cu Kα radiation at 40 kV, with a 0.02° scanning range from 10° to 80°, a step size of 0.02°, and a scanning rate of 8°/min. The surface morphology of the samples was examined using a Hitachi S-4800 scanning electron microscope (SEM, Hitachi, Tokyo, Japan). An accelerating voltage of 5–10 kV was applied during the analysis. The specific surface area and pore structure of the AS sample were determined using a 3Flex America Micromeritics analyzer (Micromeritics, Norcross, GA, USA). Prior to analysis, the samples were vacuum degassed at 300 °C for 12 h. The analysis was performed at 77 K. The elemental composition (carbon, hydrogen, and nitrogen) of AS was determined using a VARIO-EL III CHNS-O Analyzer (Elementar Analysensysteme GmbH, Langenselbold, Germany).

### 2.2. Sorption of Zinc Ions

Batch sorption experiments were conducted to thoroughly investigate the affinity of the apricot stone sorbent for Zn(II) ions. These investigations systematically examined the individual effects of the solution pH, sorbent mass, initial Zn(II) ion concentration, and contact time. The experiments were performed in conical flasks containing 50 mL of aqueous Zn(II) solution and 50 mg of sorbent. The mixtures were agitated at 250 rpm using an orbital shaker, maintained at 25 °C, and allowed to interact for 60 min at the native solution pH.

To evaluate the impact of pH, the initial solution values were adjusted between 2 and 6 using 0.1 M NaOH or 0.1 M HNO_3_ before the addition of the sorbent and subsequent agitation. The effect of sorbent dosage was assessed by adding varying amounts of apricot stone powder (6–50 mg) to the shaken Zn(II) ion solutions. These specific dosage experiments were conducted for 60 min at 25 °C, and the native solution pH.

For isothermal studies, the initial Zn(II) ion concentrations were set between 5 and 40 mg/L, with experiments performed under equilibrium conditions. Langmuir, Freundlich, and Sips isotherm models were employed to fit and assess the isotherm data. The sorption kinetics were investigated by monitoring Zn(II) ion uptake over contact times ranging from 0 to 30 min. The kinetic behavior was theoretically examined using standard models: pseudo-first-order and pseudo-second-order, where all parameters were determined via linear regression analysis processed through Origin 8.5 software [24]. The accuracy of the calculated sorption capacities relative to experimental results was evaluated using the Root Mean Squared Error (RMSE) to quantify the deviation, as defined by the following expression:

(1) $$RMSE=\sqrt{\frac{1}{n}}\sum_{i=1}^{n}\left|q_{e,exp}-q_{e,cal}\right|$$

Following the sorption experiments, all samples were filtered using a 0.45 μm membrane filter (Agilent Technologies, Waldbronn, Germany). The residual Zn(II) ion concentrations in the filtrates were quantified by measuring the concentration at 213.856 nm using Inductively Coupled Plasma-Optical Emission Spectrometry (ICP-OES) (Thermo Scientific iCAP 6500, Waltham, MA, USA).

Sorbent regeneration studies were performed to assess the reusability across multiple cycles. The Zn-loaded sorbent was initially washed with a 50/50 (*v*/*v*) ethanol/1 M acetic acid mixture for 2 h to facilitate Zn(II) ion desorption, followed by several rinses with deionized water. The regenerated sorbent was then dried at 60 °C for 1 h before being recontacted with a 20 mg/L Zn(II) solution, following previously described batch sorption conditions. This complete sorption–desorption cycle was repeated four consecutive times to evaluate the regeneration efficiency.

Understanding the efficiency and capacity of sorbents is crucial for predicting and optimizing sorption processes in various environmental and industrial contexts. The sorption capacity (q_e_, mg/g), which represents the maximum amount of sorbate that a sorbent can take up under specified conditions, was calculated using the following equation:

(2) $$q_{e}=\frac{(C_{0}\;-\;C_{e})\times V}{m}$$

The removal efficiency (ADS, %), expressed as a percentage, quantifies the effectiveness of a sorbent in eliminating a target substance from a liquid or gaseous phase, and is calculated as:

(3) $$ADS\;(\%)=\frac{\left(C_{o}\;-\;C_{e}\right)}{C_{0}}\times 100$$

In these equations, C_0_ and C_e_ denote the initial and equilibrium (or final) concentrations of the Zn(II) ions (in mg/L), respectively. V represents the solution volume (L), and m is the mass of the apricot stone sorbent (in g). All sorption experiments were performed in triplicate, and the average values are presented. The standard deviation was less than 10%.

### 2.3. Ecotoxicity

The ecotoxicity of both the pristine and metal-ion-loaded sorbents was assessed using a phytotoxicity test, specifically a seed germination test. This test was conducted in accordance with a combination of established protocols, including the OECD 208 terrestrial plant growth test and the ISO 11269-2 International Standard [25,26]. The experimental protocol involved placing filter paper in Petri dishes, then adding five radish seeds to each dish. Radish seeds were selected because they are among the 10 plant species recommended by the standard, primarily for their rapid germination rate and robust root growth. For the control group, 1 mL of distilled water was added to each Petri dish. For the treatment groups, 1 mL of distilled water containing 1 mg of either the pristine sorbent or the metal-ion-loaded sorbent was added to each dish. All Petri dishes were then incubated at 25 °C, and seed germination was carefully monitored over 7 days. After this incubation period, seedlings were collected to measure both root and shoot length accurately. The Germination Index (GI) was calculated using the following equation:

(4) $$GI(\%)=\frac{\left(GSS\times ARLS\right)}{\left(GSC\times ARLC\right)}\times 100$$

where GSS is the germinated seed in the sample, ARLS is the average root length in the sample, GSC is the germinated seed in the control, and ARLC is the average root length in the control. The germination assay was performed in triplicate, and the average values are presented. The standard deviation was up to 5%.

## 3. Results and Discussion

### 3.1. Biosorbent Characteristics

**Figure 1:**
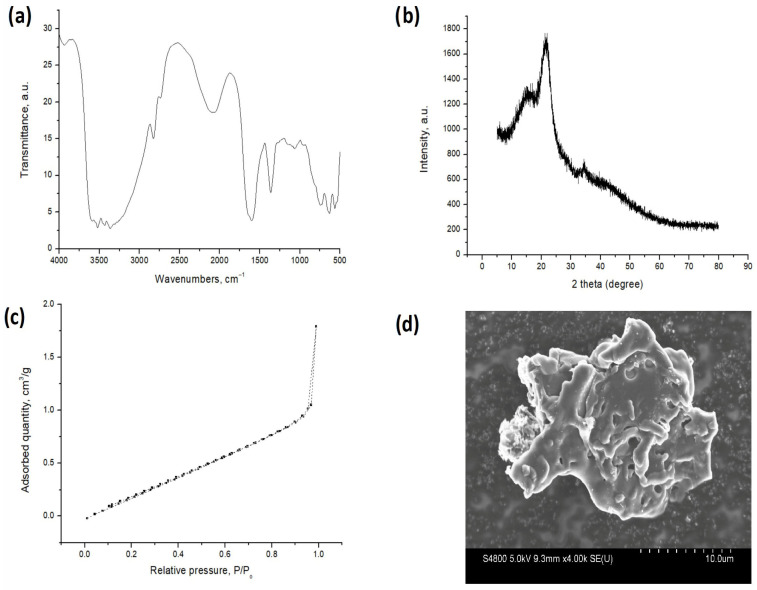
The characterization of apricot stone powder: (**a**) FTIR analysis, (**b**) XRD diffractogram, (**c**) Nitrogen sorption–desorption isotherm, and (**d**) SEM micrograph [34].

The FTIR spectrum (Figure 1) confirms the lignocellulosic nature of the apricot stone powder, displaying characteristic bands for O–H stretching (3400 cm^−1^), aliphatic C–H stretching (2900 cm^−1^), and C–O functionalities (1000–1100 cm^−1^) [27,28]. Peaks associated with hemicellulose and lignin are evident through C=O stretching (1700 cm^−1^) and aromatic C=C vibrations (1500–1600 cm^−1^) [29,30]. The abundance of these oxygen-containing functional groups—specifically hydroxyl, carbonyl, and carboxyl moieties provides the necessary active sites for Zn(II) complexation and ion exchange. The high oxygen (49.4%) and carbon (45.8%) content obtained by elemental analysis confirms that the raw apricot stone powder is a lignocellulosic material, signifying a rich concentration of oxygen-containing surface functional groups like hydroxyl, carboxyl, and carbonyl, that can serve as the primary active sites for metal ion coordination and chelation.

The XRD pattern provides information about the material’s crystalline structure. The dominant broad peak observed around the 22–25° region is characteristic of an amorphous or highly disordered carbonaceous structure, which is typical of biomass-derived materials or biochars produced at moderate temperatures. The absence of sharp, distinct peaks indicates that the apricot stone powder lacks significant crystalline phases, such as graphite or specific mineral forms [31].

The nitrogen sorption–desorption isotherm obtained for the apricot stone powder displays a Type II profile with a discernible hysteresis loop, which is characteristic of non-porous or microporous materials that may also possess some mesoporosity. The initial low uptake at low relative pressures (P/P_0_) suggests limited microporosity. However, a significant increase in the adsorbed quantity at higher relative pressures (P/P_0_ > 0.8) indicates a considerable macropore volume and/or inter-particle condensation within the sample. The Brunauer–Emmett–Teller (BET) specific surface area of the apricot stone powder was determined to be 1.71 m^2^/g. The total pore volume was calculated to be 0.0027 cm^3^/g, and the average pore diameter was found to be 6.5 nm. This average pore diameter falls squarely within the mesoporous range (2–50 nm), reinforcing the observations from the isotherm shape that the material primarily consists of mesopores rather than extensive micropores. While the specific surface area is relatively low compared to that of highly porous activated carbons, the accessible mesoporous network is advantageous for metal ion uptake, as it provides wide diffusion channels that minimize mass-transfer resistance and ensure high accessibility to active surface sites. The values obtained from the nitrogen sorption–desorption analysis align well with reported data for raw apricot shells, stones, and other lignocellulosic biomaterials of a similar nature [22].

The SEM image reveals the surface morphology and particle shape of the apricot stone powder on a 10.0 μm scale. The image shows particles with irregular, fragmented, and angular shapes, which is consistent with a material that has undergone grinding and milling. The surface appears rough and uneven, with visible crevices and potentially larger pores or channels. This irregular and rough morphology contributed to the overall surface area, providing numerous physical sites and pathways that facilitated the sorption of pollutants onto the sorbent material. Similar morphologies have been reported in the literature for other lignocellulosic biomass materials, such as groundnut shells [32], cassava bagasse [33], and pistachio shells [31].

### 3.2. Zinc Removal from Water

#### 3.2.1. The Influence of pH and Biosorbent Mass

**Figure 2:**
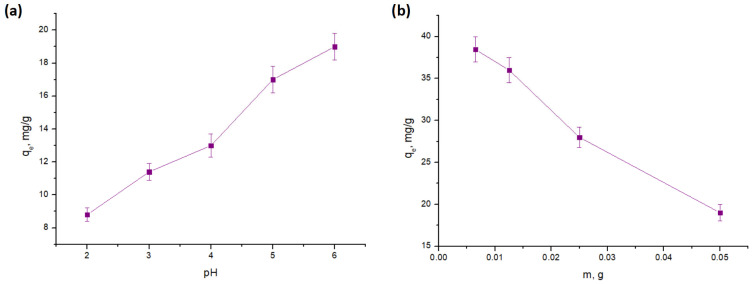
The influence of: (**a**) pH and (**b**) mass of sorbent on the sorption capacity of Zn(II) ions from aqueous solution by apricot stone.

The impact of initial solution pH on Zn(II) uptake is shown in Figure 2a. Sorption efficiency increased significantly from pH 2 (approx. 8.5 mg/g) to pH 6 (approx. 19 mg/g), where removal exceeded 90%. At low pH (2–3), high H^+^ concentrations limit Zn(II) uptake through intense competition for active sites and the protonation of surface functional groups, which creates a repulsive electrostatic environment [35]. Conversely, as pH increases toward 6, the reduction in H^+^ competition and the deprotonation of carboxyl and hydroxyl groups (forming -COO- and -O-) enhance electrostatic attraction and complexation with Zn(II) cations [36,37]. This trend is consistent with PZC results (see Table 2) and previous studies on Cu(II) and Fe(II) sorption using similar lignocellulosic matrices [22,23].

**Figure 3:**
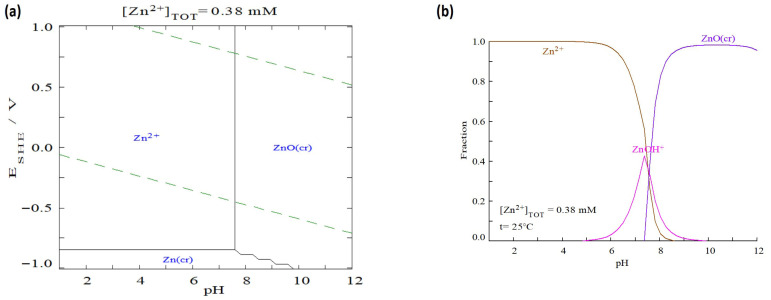
(**a**) Pourbaix (Eh–pH) diagram for zinc (Zn) species. Dashed lines represent the upper (oxygen generation) and the lower (hydrogen formation) stability limits of water, (**b**) Distribution of zinc (Zn) species as a function of pH.

It is important to underline that the experiments were not conducted at pH values above 6. This decision was informed by the thermodynamic stability of zinc in aqueous solutions; as illustrated in the E_H_-pH and speciation diagrams (see Figure 3), Zn exists predominantly as the free Zn^2+^ cation below pH 7.0. However, the formation of the monovalent ZnOH^+^ species begins near pH 6.5, followed rapidly by the precipitation of insoluble crystalline zinc oxide (ZnO(cr)) as the pH rises toward 7.5. Such precipitation would confound the true sorption process, making it difficult to accurately assess the sorbent’s performance [1]. Therefore, the maximum sorption observed at pH 6 represents the optimal condition within the range in which genuine sorption dominates the removal process.

The effect of sorbent dosage on Zn(II) removal is shown in Figure 2b. As the apricot stone mass increased from 0.006 g to 0.05 g, the removal percentage rose from 25% to 95%, driven by the greater availability of active sorption sites. Conversely, the equilibrium sorption capacity decreased from 38 mg/g to 19 mg/g over the same range. This decline is attributed to the underutilization of active sites at higher dosages, where the fixed number of Zn(II) ions is insufficient to saturate the surplus of available surface sites, leading to a decrease in the concentration gradient and the amount of metal adsorbed per unit mass [5,38].

Furthermore, the initial concentration of Zn(II) ions available per unit mass of the sorbent is effectively lower. This can reduce the driving force (concentration gradient) for sorption, making the utilization of each sorption site less efficient. Previous studies on the sorption of metal ions onto biomass/agricultural residues have shown the same trend [5,39,40,41].

#### 3.2.2. Kinetic and Isotherm Models

**Figure 4:**
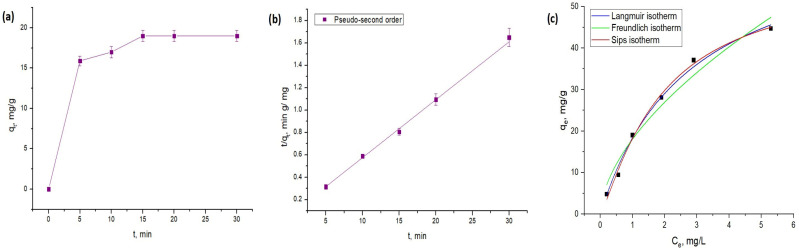
(**a**) Influence of contact time on sorption capacity, (**b**) pseudo-second order kinetic model, (**c**) isotherm models.

Figure 4 presents a comprehensive kinetic and isotherm analysis of Zn(II) ion sorption onto apricot stone powder, aimed at elucidating the sorption mechanism and rate-limiting steps. Figure 3a illustrates the rapid initial uptake of Zn ions by apricot stone powder. Both the sorption capacity and the sorption percentage increase sharply within the first 10–15 min of contact time. This rapid initial phase suggests a high availability of active binding sites on the sorbent surface and strong sorption driving forces. Following this initial surge, the sorption rate significantly slows down, reaching equilibrium at approximately 20 min. Beyond this point, there is no substantial increase in either q_e_ or ADS%, indicating that the available sorption sites are largely saturated and that the system has reached dynamic equilibrium. The high sorption percentage (95%) achieved within a relatively short time highlights the efficiency of the apricot stone powder as a sorbent for Zn(II) ions. This performance surpasses those of several other biomass sorbents for the removal of Zn(II) ions, as documented in the literature. For instance, watermelon peel requires 60 min, sugarcane bagasse for 120 min, cassava bagasse for 180 min, peat and durian peel for 240 min, jackfruit peel for 350 min, and banana/orange peels for 1440 min for comparable Zn ion removal [6,33,35,42,43,44,45].

Two standard kinetic models, the pseudo-first-order and pseudo-second-order models, were applied to the experimental data to elucidate the rate-limiting steps in Zn(II) ion uptake. The low R^2^ value and significant deviation in sorption capacity (RMSE = 10.9) indicate that the pseudo-first-order model fails to accurately describe the sorption kinetics of Zn(II) ions onto apricot stones. In contrast, the pseudo-second-order kinetic model provides an excellent fit to the experimental data, yielding R^2^ of 0.99 and a RMSE of 0.03. Crucially, the theoretical equilibrium capacity derived from this model matches the experimental value, confirming that the pseudo-second-order model accurately captures the system’s kinetic behavior. The observed fit of the pseudo-second-order kinetic model is well supported by numerous scientific studies on the sorption of heavy metal ions onto various agricultural wastes [6,43,46,47].

The Langmuir, Freundlich, and Sips (Langmuir–Freundlich) isotherm models were employed to understand the equilibrium behavior and nature of the interaction between Zn(II) ions and the apricot stone sorbent. The obtained parameters are presented in Table 3. Among the tested isotherm models, the Sips model provides the best fit to the data (as evidenced by the lowest chi parameter and highest R^2^), followed closely by the Langmuir model. The superior fit of the Sips model, with its heterogeneity parameter (n = 1.2) slightly above 1, suggests that while sorption is largely monolayer-driven, the apricot stone surface might exhibit some degree of heterogeneity. The maximum sorption capacity predicted by the Sips model (q_max_ = 58.2 mg/g) highlights the significant potential of apricot stone powder as an effective sorbent for Zn(II) ion removal from aqueous solutions.

**Figure 5:**
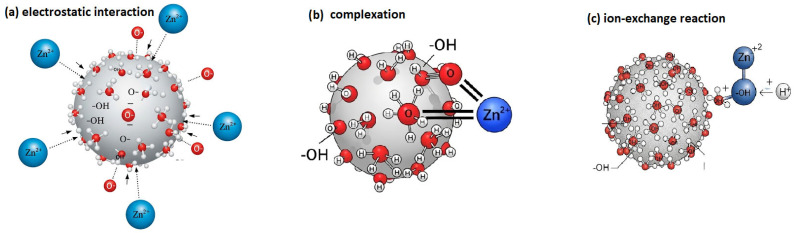
The proposed mechanism of Zn(II) ion sorption onto apricot stone powder.

The sorption of Zn(II) onto apricot stone is a multi-faceted process governed by surface heterogeneity and chemical interactions. The Sips isotherm fit indicates that the biomass surface contains a distribution of binding sites with varying affinities, likely involving hydroxyl and carboxyl groups in different chemical environments. The sorption mechanism is governed by the biomass’s surface charge, as evidenced by its point of zero charge (PZC) of 5.5. At the optimal working pH of 6, the solution pH exceeds the PZC, resulting in a net negative surface charge due to the deprotonation of functional groups. This creates a favorable environment for the electrostatic attraction of Zn ions, which facilitates subsequent chemisorption via ion exchange (replacing H+ protons) or surface complexation with oxygen-donating moieties (Figure 5). These complementary surface-chemical findings reinforce the conclusion that the process is not merely physical accumulation but is dominated by chemical interactions between the metal ions and the biomass matrix.

#### 3.2.3. Test with Industrial Wastewater and Reusability

**Figure 6:**
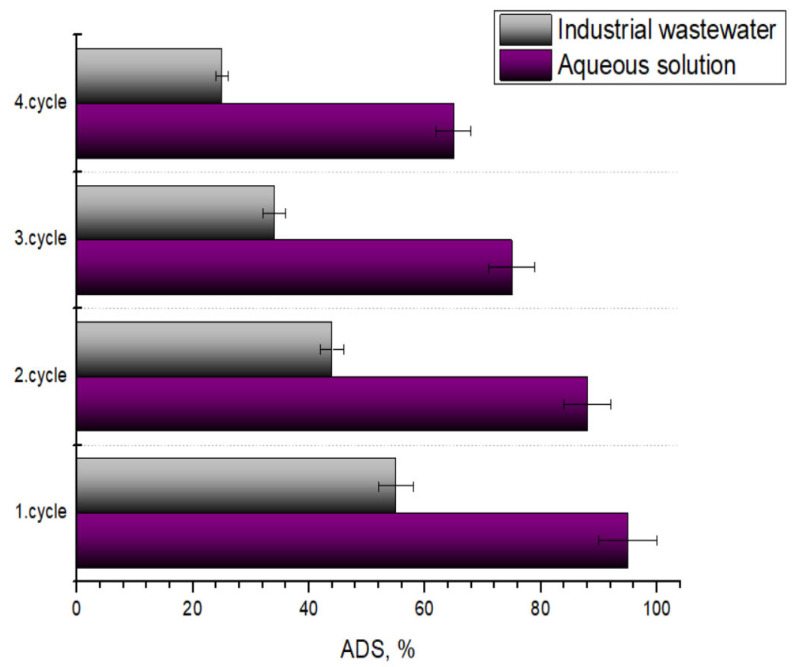
Comparison of apricot stone efficiency to remove Zn(II) ions in aqueous solution and real wastewater during several sorption–desorption cycles.

Figure 6 compares the performance of the apricot stone sorbent for removing Zn ions from aqueous solutions and industrial wastewater sourced from the zinc galvanization industry. The industrial wastewater used in this study, sourced from the galvanization industry, contained Zn(II) ions at 25 ppm and had a pH of 6.8. To evaluate the material under realistic conditions, sorption was performed using the same parameters as in the aqueous batch studies (50 mg of sorbent in 50 mL of solution for a 15 min contact time), but without pH modification. Additionally, it assesses the sorbent’s reusability over four consecutive sorption–desorption cycles in both scenarios. Each desorption cycle was performed in ethanol + 1 M acetic (50/50 *v*/*v*) for 2 h. In aqueous solution, the sorbent achieves an impressive initial removal efficiency (1st cycle) of approximately 95% (q_e_ = 19 mg/g), whereas in industrial wastewater, the 1st-cycle removal efficiency drops significantly to around 55% (q_e_ = 13.75 mg/g). This substantial reduction is directly attributable to the inherent complexity of galvanization wastewater, which contains various inorganic salts, acids, bases, and a range of organic additives (such as brighteners and complexing agents) used in the galvanization process. These co-contaminants can compete directly with Zn(II) ions for the sorbent’s available active binding sites. Furthermore, they can interfere with the sorption process by altering the solution’s ionic strength.

Regarding sorbent reusability, a consistent trend of decreasing sorption efficiency with each subsequent cycle is observed in both cases, indicating a decline in the sorbent’s regenerative capacity. Although the initial removal in aqueous solutions is very high (95%), the removal efficiency drops to approximately 60–65% by the 4th cycle. The drop in efficiency across cycles is even more pronounced for industrial wastewater. Starting from 55% in the 1st cycle, the removal efficiency plummets to approximately 20% by the 4th cycle. This accelerated decline in performance is likely due to fouling by co-contaminants. Other components in industrial wastewater may be irreversibly adsorbed onto the sorbent, permanently blocking active sites and hindering regeneration.

In conclusion, apricot stone powder demonstrates promising initial performance for Zn(II) ion removal, especially in controlled aqueous environments. However, its efficiency is notably reduced in complex industrial wastewater, owing to competitive sorption and matrix effects. Furthermore, although the sorbent exhibits some reusability, its regeneration efficiency decreases significantly over consecutive cycles, particularly when treating industrial wastewater. Hence, while the developed apricot stone adsorbent shows high efficiency in model solutions, its application in real galvanic wastewater must be viewed within the context of multi-component treatment systems. Galvanic effluent typically undergoes primary treatment via chemical precipitation and coagulation-flocculation to remove the bulk of heavy metal concentrations. The bio-sorbent described in this study is highly promising as a tertiary polishing agent. In this role, it can effectively sequester trace Zn(II) ions that persist after primary treatment, ensuring the final discharge meets stringent environmental regulations.

#### 3.2.4. Ecotoxicity

Saturated sorbents containing heavy metals can become a new source of pollution if not disposed of properly. Therefore, an ecotoxicity test is crucial for assessing the environmental risk posed by spent sorbent and ensuring it does not harm living organisms or ecosystems upon release. The germination test is the first in vitro screening method to assess the potential harm a substance may cause in soil. This test is significant for assessing the phytotoxicity of a sorbent, which is the degree to which it is toxic to plants.

**Figure 7:**
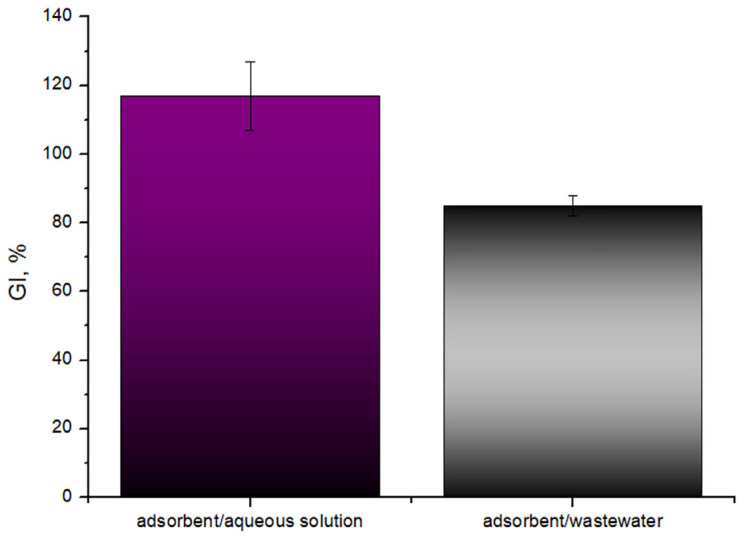
Germination index of apricot stone-Zn(II) ions materials.

Figure 7 presents a comparative analysis of the phytotoxicity of the apricot stone sorbent, specifically evaluating its impact on radish seed germination after treatment with Zn-contaminated aqueous solutions and industrial wastewater. The results are expressed as the Germination Index (GI, %), a key indicator of plant growth ecotoxicity. After treatment with an aqueous Zn(II) solution, the apricot stone sorbent shows a remarkably high Germination Index of approximately 115–120%. A GI value above 100% indicates that the treated solution, or the sorbent itself, not only did not inhibit plant growth but potentially has a beneficial or stimulating effect on radish seed germination and early growth compared to the control. This is a highly favorable outcome, suggesting that the apricot stone sorbent effectively removes toxic Zn ions from a simple aqueous solution to a non-toxic level, and may even promote plant development. This also implies that the sorbent material itself, after treatment, is not inherently phytotoxic. In contrast, after treating the industrial wastewater, the apricot stone sorbent exhibits a lower Germination Index of approximately 80–85%. Although still indicating a relatively good level of germination (above 65% is generally considered non-toxic) [48,49], this value is significantly lower than that observed for the aqueous solution treatment. The reduction in GI of the wastewater-treated sorbent can be attributed to sorbent-bound contaminants, which are related to the complex nature of industrial wastewater. It is possible that some components from industrial wastewater, other than Zn, are adsorbed onto the apricot stone sorbent itself, and then subsequently leach out or interact with the seeds during the germination test, leading to reduced growth.

It is important to highlight that although GI is lower after treating complex industrial wastewater, the apricot stone sorbent and any residual contaminants associated with it do not pose a threat to plant growth. This aligns well with established sorption results, given that the sorbent adsorbs approximately 12–15 ppm of Zn(II) ions from industrial wastewater. This level of Zn does not pose a risk to the plants. In fact, the maximum allowable Zn limit in soil, before it is considered toxic, is approximately 300 ppm [50]. From a nutritional perspective, Zn is an essential micronutrient for plants that plays a vital role in numerous physiological processes. It is a key component of various enzymes, participates in carbohydrate and protein metabolism, and is crucial for chlorophyll formation and proper growth regulation. Indeed, some plants may even exhibit stunted growth or fail to thrive in the absence of sufficient Zn. Therefore, the presence of Zn in the discarded sorbent, within safe limits, could even be beneficial in certain soil environments where this essential nutrient is lacking. Hence, the apricot stone sorbent can be considered an efficient and promising material for real-world wastewater treatment applications. The lack of phytotoxicity indicates that the Zn-loaded sorbent is biologically compatible, suggesting its potential for safe soil application if it meets further local soil regulatory standards.

#### 3.2.5. Comparison with Literature

The sorption performance of the apricot stone as a sorbent for Zn(II) ion removal was compared with that of other biomass sorbents previously reported in the literature (Table 4). The results indicated that raw apricot stone, with a maximum sorption capacity of 58.2 mg/g and an adequate contact time of only 15–30 min, significantly outperforms many commonly used agricultural wastes. Compared to sorbents such as natural bagasse, wheat straw, and banana peel, apricot stone demonstrated a more than tenfold increase in sorption capacity and drastically reduced equilibrium time. Even sorbents with higher performance, such as jackfruit peel, neem bark, and rapeseed waste, are substantially less effective in terms of both capacity and kinetics. These attributes distinguish apricot stone powder as a highly competitive, unmodified sorbent, particularly for high-throughput systems where both rapid kinetics and high capacity are essential.

## 4. Conclusions

This study demonstrates that apricot stone powder is an effective biosorbent for Zn(II) removal, characterized by rapid kinetics and a heterogeneous surface binding mechanism best described by the Sips isotherm. While the material shows high efficiency in synthetic solutions, its performance significantly decreases in complex galvanic wastewater due to matrix effects and ion competition. Furthermore, ecotoxicological assays indicate no phytotoxicity, suggesting that the spent sorbent is biologically compatible under the tested conditions. These findings position apricot stone as a high-potential, renewable resource for waste valorization, offering a sustainable and scalable framework for integrating agricultural byproducts into modern wastewater treatment strategies.

## Figures and Tables

**Table 1:** Characteristics of galvanized wastewater.

pH	6.5
Cations	mg/L
K	160
Ca	111
Na	62.7
Mg	25.6
Zn	25
Co	0.24
Ni	0.069
Mn	0.045
Anions	mg/L
Cl^−^	18.3
SO_4_^2−^	11.2
NO_3_^−^	0.88

**Table 2:** Textural, elemental parameters, and PZC of apricot stone powder.

BET, m^2^/g	1.72
Vtot, cm^3^/g	0.0027
D, nm	6.5
C, %	45.8
H, %	4.79
N, %	0.05
O, %	49.4
PZC	5.5

**Table 3:** Kinetic and isotherm model parameters for the sorption of Zn(II) ions onto apricot stone powder (n = 3).

Pseudo-first kinetic model	
R^2^	0.73
k_1_, 1/min	0.12 ± 0.005
q_e,cal_, mg/g	7.96 ± 0.3
RMSE	10.91
Pseudo-second kinetic model	
R^2^	0.99
k_2_, g/mg min	0.07 ± 0.004
q_e,cal_, mg/g	18.9 ± 0.8
RMSE	0.03
Langmuir isotherm model	
R^2^	0.99
Χ^2^	2.17
Q_max_, mg/g	69.6 ± 2
K_L_ (L/mg)	0.359 ± 0.02
Freundlich isotherm model	
R^2^	0.956
Χ^2^	10.67
n	1.83 ± 0.09
K_F_, (mg/g) (L/mg)^1/n^	18 ± 0.9
Sips isotherm model	
R^2^	0.99
Χ^2^	1.79
n	1.2 ± 0.05
K_F_, (mg/g) (L/mg)^1/n^	0.455 ± 0.02
Q_max_, mg/g	58.2 ± 2.5

**Table 4:** Comparison of apricot stone with other biomass sorbents for the removal of Zn(II) ions from aqueous solution.

Sorbent	q_e_, mg/g	Time, min	Reference
Natural Sugarcane bagasse	0.40	120	[44]
Wheat straw	3.25	30	[27]
Lemon peel	5.03	15	[39]
Cassava bagasse	5.65	300	[33]
Banana peel	5.8	1440	[45]
Peat	5.84	240	[6]
Watermelon rind	6.85	60	[51]
Jackfruit peel	9.37	350	[43]
Coffee husk	11.11	30	[52]
Neem bark	13.29	300	[53]
Rapeseed waste	13.86	400	[1]
Apricot stone	58.2	15–30	This work

## Data Availability

The data presented in this study are confidential.
